# Feature visualization and classification for the discrimination between individuals with Parkinson’s disease under levodopa and DBS treatments

**DOI:** 10.1186/s12938-016-0290-y

**Published:** 2016-12-30

**Authors:** Alessandro R. P. Machado, Hudson Capanema Zaidan, Ana Paula Souza Paixão, Guilherme Lopes Cavalheiro, Fábio Henrique Monteiro Oliveira, João Areis Ferreira Barbosa Júnior, Kheline Naves, Adriano Alves Pereira, Janser Moura Pereira, Nader Pouratian, Xiaoyi Zhuo, Andrew O’Keeffe, Justin Sharim, Yvette Bordelon, Laurice Yang, Marcus Fraga Vieira, Adriano O. Andrade

**Affiliations:** 1 Postgraduate Program in Electrical and Biomedical Engineering, Faculty of Electrical Engineering, Centre for Innovation and Technology Assessment in Health (NIATS), Federal University of Uberlândia, Uberlândia, Brazil; 2 Faculty of Mathematics, Federal University of Uberlândia, Uberlândia, Brazil; 3 Department of Neurosurgery, University of California, Los Angeles, USA; 4 Department of Neurology, University of California, Los Angeles, USA; 5 Neurology and Neurological Sciences, Stanford University, Stanford, CA USA; 6 Bioengineering and Biomechanics Laboratory, Federal University of Goiás, Goiânia, Brazil

**Keywords:** Parkinson’s disease, Deep brain stimulation, Levodopa, Inertial sensors, Electromyography, Discriminant analysis

## Abstract

**Background:**

Over the years, a number of distinct treatments have been adopted for the management of the motor symptoms of Parkinson’s disease (PD), including pharmacologic therapies and deep brain stimulation (DBS). Efficacy is most often evaluated by subjective assessments, which are prone to error and dependent on the experience of the examiner. Our goal was to identify an objective means of assessing response to therapy.

**Methods:**

In this study, we employed objective analyses in order to visualize and identify differences between three groups: healthy control (N = 10), subjects with PD treated with DBS (N = 12), and subjects with PD treated with levodopa (N = 16). Subjects were assessed during execution of three dynamic tasks (finger taps, finger to nose, supination and pronation) and a static task (extended arm with no active movement). Measurements were acquired with two pairs of inertial and electromyographic sensors. Feature extraction was applied to estimate the relevant information from the data after which the high-dimensional feature space was reduced to a two-dimensional space using the nonlinear Sammon’s map. Non-parametric analysis of variance was employed for the verification of relevant statistical differences among the groups (*p* < *0.05*). In addition, K-fold cross-validation for discriminant analysis based on Gaussian Finite Mixture Modeling was employed for data classification.

**Results:**

The results showed visual and statistical differences for all groups and conditions (i.e., static and dynamic tasks). The employed methods were successful for the discrimination of the groups. Classification accuracy was 81 ± 6% (mean ± standard deviation) and 71 ± 8%, for training and test groups respectively.

**Conclusions:**

This research showed the discrimination between healthy and diseased groups conditions. The methods were also able to discriminate individuals with PD treated with DBS and levodopa. These methods enable objective characterization and visualization of features extracted from inertial and electromyographic sensors for different groups.

## Background

Parkinson’s disease (PD) is a neurodegenerative disorder with progressive motor symptoms [1]. PD affects approximately 3% of the population over 65 years old [2] and is associated with a severe loss in function of dopaminergic neurons within the substantia nigra pars compacta [3]. The primary symptoms of PD are tremors (oscillatory movements), bradykinesia (slow movement) and rigidity (increased muscular tone) [3–9]. PD can have significant negative impacts in several aspects of patients’ quality of life, including those associated with physical and social functioning, such as affecting patient’s ability to drink, eat and write [10–12].

There is a growing consensus that states that PD can manifest with different patterns [13]. Distinct subtypes manifest different symptom patterns, such as tremor dominant (TD) type and postural instability and gait difficult (PIGD) type (bradykinesia and rigidity) [13, 14]. These subtypes are associated with different patterns of onset and rate of disease progression [15, 16]. Moreover, different PD subtypes have been linked to different genetic patterns [14].

Ensuring a correct diagnosis is critical for prognostic and therapeutic reasons and also for clinical, epidemiological, and pharmacological studies [17]. Despite all the advances obtained in neuroimaging and genetics, the diagnosis of PD remains primarily clinical [17]. Severity of disease is most often evaluated with the subjective Unified Parkinson’s Disease Rating Scale (UPDRS) [18], which is composed of four parts: Part I (non-motor experiences of daily living), Part II (motor experiences of daily living), Part III (motor examination) and Part IV (motor complications). There are a number of alternative rating scales that are used for the evaluation of motor impairment and disability in patients with PD, but these scales have not been evaluated for validity and reliability [19]. Due to the subjective nature of these assessments and the need for improving the diagnosis and treatment efficacy, studies must be performed to provide feedback for neurologists during clinical evaluation of patients, reducing the time and effort required to achieve optimal outcomes and improving the treatment.

Regarding available treatments for PD, the drug levodopa is the gold standard [20]. Since the discovery that dopamine loss is associated with PD, this medication is recognized as the most effective drug for PD treatment [1, 21]. However, long-term use of the medication leads to a levodopa-induced decrease in the efficacy of motor benefits and an increase in the incidence of adverse effects, which can contribute to worsening quality of life [20, 22, 23]. Invasive treatments, such as ablative surgeries and deep brain stimulation (DBS) are also used for PD treatment, although ablative methods are now largely reserved for patients with contraindications to implantable hardware and in patients who live in countries with limited economic resources [24].

The use of DBS has been shown to improve motor symptoms in patients with advanced stages of PD that are responsive to traditional medical treatment [23]. DBS was approved by the US Food and Drug Administration to treat PD motor symptoms in 2002 [25]. Hyperactivity of the subthalamic nucleus and globus pallidus internus is thought to underlie the pathophysiological mechanism of PD, making these basal ganglia regions the most commonly targeted sites for DBS [25]. DBS is applied in patients only if the symptomatic benefits are greater than the possible surgical risks and if DBS is likely to reduce overall symptomatology more effectively than drug therapy alone [12]. When optimized, DBS typically lessens motor symptoms, such as limb rigidity, akinesia, tremor and bradykinesia [23]. Regarding neuropsychological measurements after DBS surgery, some studies found decrease in cognitive functions [26] due to the development of apathy in some subjects [27].

A number of studies have compared outcomes between DBS and best medical management [12, 22, 23, 27–33]. Most of current studies employ subjective scales to measure the differences in the methods of treatment. The review of Xie et al. [23] evaluated differences between DBS and medication treated groups, finding that individuals treated with DBS fared better than patients treated with medications with respect to motor complications as assessed by the UPDRS. However, the authors stated that due to the small number of studies, results must be prudently evaluated. Deuschl et al. [22] evaluated 156 patients under 75 years old with advanced Parkinson’s disease and severe motor symptoms. They found that, according to subjective scales, DBS provided better outcomes in social and motor results. It was also emphasized that the group that received neurostimulation is more susceptible to serious adverse effects, including fatal cerebral hemorrhage. De Gaspari et al. [33] also evaluated patients with medication and patients with DBS. It was found that both groups revealed significant improvements in the motor function. However, regarding neuropsychiatric scales, DBS seemed to be associated with significant worsening, resulting in long term behavioral problems for some patients.

Despite numerous studies comparing outcomes across groups, we could not identify in our literature review the systematic use of an objective method for comparing and visualizing the possible differences of individuals submitted to distinct treatment approaches. Furthermore, most studies do not contrast the motor behavior of healthy subjects with that of PD patients submitted to different treatments. As it is known that subjects with DBS show improvements in motor behavior when compared with subjects treated with medication [22, 23], an automatic classifier could theoretically be developed to compare these groups and show whether patients treated with DBS objectively demonstrate the expected improvements. Furthermore, an objective discrimination between healthy subjects and PD patients treated with medication could provide us with parameters that may be related to the efficacy of the treatment, making it possible to improve medical management of PD [22].

In order to capture patterns of movements and objectively be able to analyze PD motor behaviors, wearable devices with built-in sensors are presented in several studies [2, 4, 5, 34–37]. Most of the devices consist of wearable systems containing inertial sensors, such as gyroscopes and accelerometers and others use electromyographic sensors in order to collect electrical activity from the muscles of the affected limb.

This research describes the use of a system composed of a customized glove with built-in inertial sensors (accelerometer, gyroscope and magnetometer) and electromyographic sensors, used for the characterization of wrist motor symptoms in three groups of subjects. The first one is composed of PD patients treated with DBS, the second is composed of PD patients treated with levodopa and the third is composed of healthy subjects, with no movement disorders. Each subject performed a set of static and dynamic tasks routinely employed by the neurologist during clinical evaluation. The aim of this study is to introduce a method for automatic classification between these groups, which can benefit our objective understanding of various treatments and improve patient management.

## Methods

### Characterization of the experimental group

This study was conducted in the Federal University of Uberlândia (UFU), Uberlândia, Brazil, and at the University of California, Los Angeles (UCLA), USA. Both institutions provided ethical approval for the experimental procedures (UCLA IRB 14-001491; CAAE 07075413.6.0000.5152).

In total, 38 subjects participated in this study. These subjects were classified as neurologically healthy individuals (S_H_ = 10), individuals with PD treated with levodopa (S_PD_ = 16), and individuals with PD treated with DBS (S_DBS_ = 12).

The inclusion criteria for the S_PD_ group were the existence of PD motor complications, treatment with levodopa and absence of DBS implant. For the S_H_ group, the inclusion criteria were the absence of movement disorder or any other neurological condition. The inclusion criteria for the S_DBS_ group were the use of DBS for the treatment of PD motor complications.

Individuals of the experimental group S_DBS_ (11 men and 1 woman aged between 59 and 76 years old) were recruited at UCLA, whereas individuals of the S_H_ group (3 men and 7 women aged between 23 and 64 years old) and S_PD_ group (6 men and 10 women aged between 47 and 92 years old) were recruited at UFU.

### Device for detecting and recording voluntary movement and tremor of the wrist

**Fig. 1:**
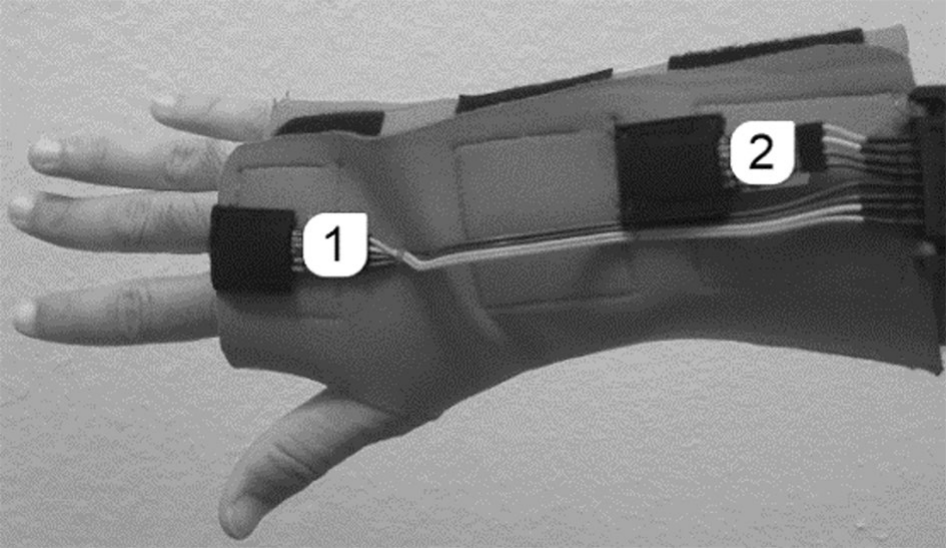
Positioning of inertial sensors. Inertial sensors embedded in the custom-made glove for tremor measurement. The unit *1* is positioned on the hand, whereas the unit *2* is positioned on the forearm

**Fig. 2:**
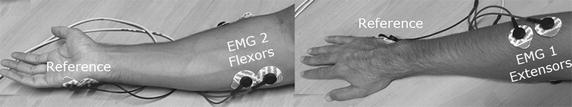
Positioning of electromyographic sensors. EMG electrode positioned at the flexor muscles of the hand and at the extensor muscles of the hand. The reference electrodes are placed on the hand

A customized glove was designed (National Industrial Property Institute, Brazil—INPI. Patent number: BR 10 2014 023282 6) including two sets of inertial sensors (Sensor 1 and Sensor 2) (Fig. 1) and two pairs of disposable electromyographic (EMG) sensors (EMG 1 and EMG 2, in Fig. 2) (diameter of 36 mm, Ag/AgCl—Meditrace 200, Tyco/Kendall, USA) that are placed on the limb of the individual. Each set of inertial sensor (L3GD20H and LSM303D, STMicroelectronics, Switzerland) is composed of three axial accelerometers (minimum sensitivity of ±2 g), gyroscopes (minimum sensitivity of ±245°/s) and magnetometers (minimum sensitivity of ±2 gauss). The approximate mass of the composite sensor is less than 1 g. The glove was designed with neoprene, a very soft and comfortable material. Due to the softness of the glove, the subjects did not feel any discomfort during the procedures. The sensors could be removed from the glove for sterilization.

EMG signals were conditioned and low-pass filtered by specific hardware (EMG System do Brasil, Brazil) to identify the signal envelope. The system was battery powered for isolation purposes.

The signals were digitized at 50 Hz, by using a microcontroller (Atmel SAM3X8E ARM Cortex-M3). The resolution of the analog to digital converter was 12 bits. Data were sent to a laptop by means of serial communication. The control and real time visualization of data acquisition was performed by customized software (TREMSEN—Precise Tremor Sensing Technology) developed in C# (Microsoft). The software was configured to handle data from up to four sets of inertial sensors (although only two sets were used in this study), two bipolar channels of EMG signals, and external pulses for synchronization and data annotation purposes (e.g., marking the beginning and end of tasks).

Data were saved in text format to be imported and processed in MatLab (MathWorks, USA).

### Definition of experimental tasks

**Fig. 3:**
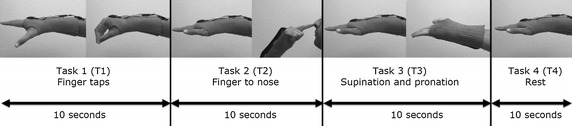
Experimental tasks. Basic sequence of executed tasks

Each participant executed the basic sequence of four tasks depicted in Fig. 3 five times. At least 30 s was allowed for rest after the end of the execution of each sequence (from tasks 1 to 4).

The four tasks performed by the volunteers were finger taps (Task 1—T1), finger to nose (Task 2—T2), supination and pronation (Task 3—T3), and rest (i.e., extended arm with no voluntary movement, Task 4—T4). Each task was performed for 10 s in sequence. In order to annotate the sequence of tasks, an external pulse was generated by pressing a pushbutton every time the subject changed the movement.

Before the beginning of the data collection with the patients we applied the so-called tremor glove standard operating procedures (TGSOP), which is an optimized protocol that involves the glove and EMG sensor positioning, and also software settings. It describes exactly what was performed before, during and after data collection. The TGSOP procedure is given below.

1. Setting up the glove system
  - Plug machine USB cable into laptop/computer;
  - Plug the external pulse generator into the External Pulse A port;
  - Plug the EMG amplifier battery into the VCC port;
  - Plug in the EMG electrodes:
    i. EMG 1—Electrodes are on wrist extensor muscles:
      1. Ask the patient to extend the wrist up in order to find the contracted muscles near lateral elbow;
    ii. EMG 2—Electrodes are on wrist flexor muscles:
      1. Ask the patient to flex the wrist down in order to find the contracted muscles near medial elbow;
    iii. Place both EMG ground electrodes near little digit, just distal to ulnar wrist (hypothenar compartment).
2. Configure the TREMSEN software (data collection software)
  - Check settings, click start and make sure that the motions are not oversaturated on recording window;
  - Write down the current settings and configurations on protocol form;
  - Inertial sensor sensitivity settings:
    i. Accelerometer: ±6 g;
    ii. Gyroscope: ±2000°/s;
    iii. Magnetometer: ±2 gauss;
3. Put glove on the tested hand—note the hand tested
4. Check with neurologist which hand has worse tremors
5. Start acquisition, have patient extend arm and rotate their hand to check for oversaturation
6. Have the patient do the following tasks for 10 s each
  d. Finger taps;
  e. Hand movements (finger to nose);
  f. Rotation of the hands (pronation and supination);
  g. Holding the arm in full extension and with no voluntary movement;
  h. Make sure to press external pulse when switching movements;
7. Tell patient to rest for 30 s
8. Repeat step 6 and 7 for more four times
9. Stop acquisition
10. Save file as patient #
  i. Check if files are saved before closing the software;
  j. It is also advised to keep a separate file of notes of any incidents that may occur during the recording, as well as relevant patient information.

## Data analysis

**Fig. 4:**
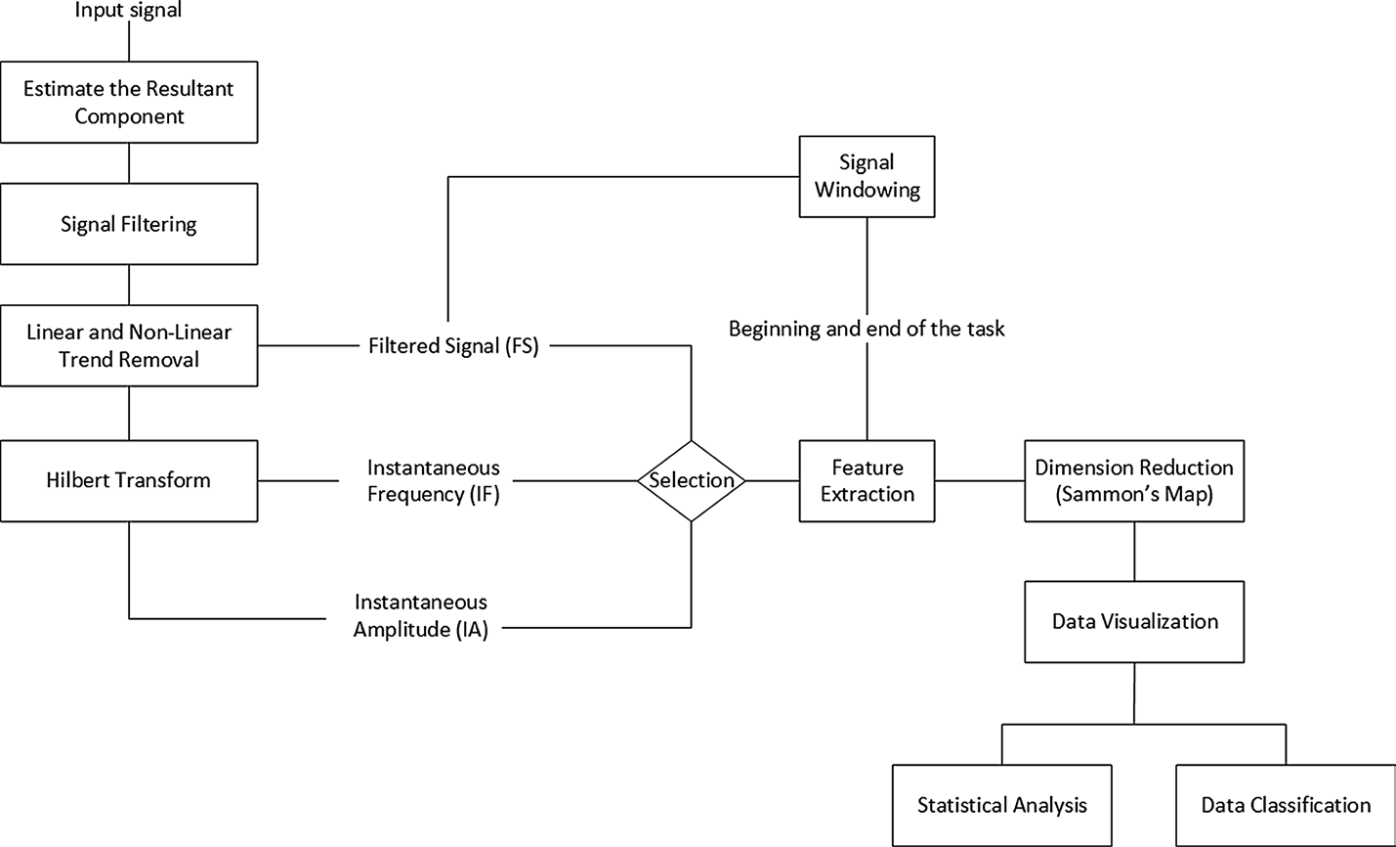
Signal processing steps. Flowchart depicting the main steps for signal processing

The flowchart in Fig. 4 summarizes the main steps for data analysis. The signals were sampled at 50 Hz.

The resultant component (step 1 in Fig. 4) of each signal was estimated as in (1), in which X, Y and Z are the individual coordinates measured by the employed sensors.

1 $$R = \sqrt {X^{2} + Y^{2} + Z^{2} }$$

The resultant component was then filtered by means of a bandpass Butterworth filter of order 4, lower cutoff frequency of 0.5 Hz and upper cutoff frequency of 25 Hz (step 2 in Fig. 4). A zero-phase forward and reverse digital IIR filtering was applied to avoid phase distortions. The main aim of this step was to eliminate the influence of undesired low frequency components over the collected signals (e.g., Gravity, Earth’s magnetic field).

In order to reduce the influence of linear and non-linear trends over the preprocessed signal, it was subtracted from its mean and also from 20th order polynomial capable of capturing nonlinear trends in the data (step 3 in Fig. 4). The main aim of this step is to guarantee that the data oscillates evenly around zero. As there is the execution of many types of tasks in a single trial the inertial sensors are subjected to distinct conditions (e.g., effect of gravity and Earth’s magnetic field), thus the resulting trend is not linear and it cannot be removed from the time-series by subtracting it from its mean. A usual method in signal processing to deal with such a condition is the application of polynomials for non-linear trend detection and removal [38]. The Hilbert transform of the preprocessed signal was employed for the estimate of the instantaneous amplitude and frequency (step 4 in Fig. 4), as described in previous work [39].

As described in [40] for an arbitrary time series, X(t), the Hilbert Transform, Y(t), is obtained from (2),

2 $$Y(t)\frac{1}{\pi }P\mathop \int \limits_{ - \infty }^{\infty } \frac{{X(t^{{\prime }} )}}{{t - t^{{\prime }} }}dt^{{\prime }}$$

where P is the Cauchy principal value defined by (3),

3 $$P\mathop \int \limits_{ - \infty }^{\infty } \frac{{X(t^{{\prime }} )}}{{t - t^{{\prime }} }}dt^{{\prime }} = \mathop {\lim }\limits_{R \to \infty } \mathop \int \limits_{ - R}^{R} \frac{{X(t^{{\prime }} )}}{{t - t^{{\prime }} }}dt^{{\prime }}$$

where R may be seen as an auxiliary variable responsible for transforming the indefinite integral into a definite one. With this definition, X(t) and Y(t) form the analytical signal, Z(t), as (4)

4 $$Z\left( t \right) = X\left( t \right) + iY\left( t \right) = a(t)e^{j\theta (t)}$$

in which (5) is the instantaneous amplitude

5 $$a\left( t \right) = [X^{2} \left( t \right) + Y^{2} ]^{{\frac{1}{2}}}$$

and (6) the instantaneous phase

6 $$\theta \left( t \right) = arctan \left( {\frac{Y(t)}{X(t)}} \right)$$

The instantaneous frequency was defined in [41] as (7)

7 $$\omega \left( t \right) = \left( {\frac{d\theta (t)}{dt}} \right)$$

In order to identify the beginning and the end of each task, a visual inspection of the signal was performed so that the annotated periods could be used for feature extraction within the regions of interest (step 5 in Fig. 4).

**Fig. 5:**
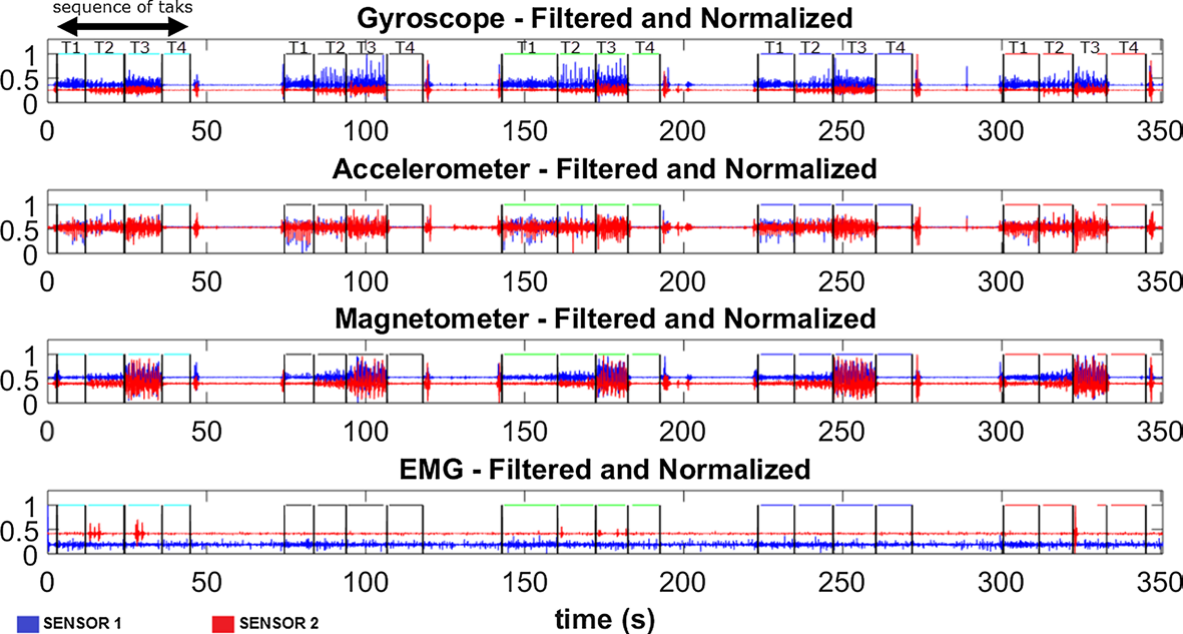
A typical example of preprocessed data. Typical results of the application of the windowing and filtering steps. The distinct tasks (T1, T2, T3 and T4) are separated by the pulses. Information regarding sensors 1 and 2 are presented in accordance to their physical location shown in Figs. 1 and 2. The external pulses are indicated by vertical lines between tasks. In periods between sequence of tasks the subject was free to execute any desired movement or to relax, thus, they are not considered in the data analysis

Figure 5 illustrates typical waveforms of resultant components for the inertial and electromyographic sensors. The periods of the sequence of executed tasks (T1, T2, T3, T4) are delimited by rectangular windows, indicating the beginning and end of each task.

Feature extraction was performed over the Filtered signal (FS), the Instantaneous Amplitude (IA) and the Instantaneous Frequency (IF), as indicated in the step 6 in Fig. 4. The following features, which are fully described in Table 1 of [42], were estimated:

**Table 1:** Success rate for the classification set

Classification												
Method	Task	S_H_ (S_H_)	S_H_ (S_PD_)	S_H_ (S_DBS_)	S_PD_ (S_H_)	S_PD_ (S_PD_)	S_PD_ (S_DBS_)	S_DBS_ (S_H_)	S_DBS_ (S_PD_)	S_DBS_ (S_DBS_)	MeanTP	StdTP
FS	1	0.85	0.11	0.04	0.11	0.79	0.10	0.12	0.18	0.70	0.78	0.05
	2	0.85	0.06	0.09	0.12	0.84	0.04	0.17	0.13	0.70	0.80	0.06
	3	0.69	0.27	0.05	0.16	0.72	0.11	0.09	0.21	0.70	0.70	0.01
	4	0.90	0.08	0.01	0.23	0.66	0.11	0.05	0.34	0.61	0.72	0.12
	Mean	0.82	0.13	0.05	0.16	0.75	0.09	0.11	0.22	0.68	0.75	0.05
	Std	0.07	0.07	0.02	0.04	0.06	0.03	0.04	0.06	0.04	0.05	0.01
IA	1	0.81	0.11	0.08	0.10	0.80	0.10	0.15	0.19	0.66	0.75	0.07
	2	0.78	0.16	0.06	0.17	0.76	0.07	0.18	0.12	0.70	0.75	0.03
	3	0.73	0.24	0.02	0.20	0.71	0.09	0.06	0.15	0.79	0.74	0.03
	4	0.93	0.04	0.03	0.12	0.76	0.12	0.11	0.35	0.54	0.74	0.14
	Mean	0.81	0.14	0.05	0.15	0.76	0.10	0.13	0.20	0.67	0.75	0.05
	Std	0.06	0.06	0.02	0.04	0.02	0.02	0.04	0.07	0.07	0.05	0.02
IF	1	0.85	0.10	0.05	0.17	0.57	0.26	0.13	0.19	0.68	0.70	0.10
	2	0.88	0.05	0.08	0.11	0.76	0.13	0.09	0.12	0.79	0.81	0.04
	3	0.79	0.16	0.05	0.24	0.56	0.20	0.10	0.13	0.77	0.71	0.10
	4	0.94	0.05	0.01	0.12	0.62	0.26	0.14	0.22	0.64	0.73	0.14
	Mean	0.86	0.09	0.05	0.16	0.63	0.21	0.11	0.17	0.72	0.74	0.08
	Std	0.04	0.04	0.02	0.05	0.07	0.05	0.02	0.04	0.06	0.06	0.01
FS-IA	1	0.84	0.12	0.04	0.09	0.80	0.12	0.13	0.18	0.69	0.77	0.06
	2	0.83	0.10	0.07	0.13	0.81	0.06	0.15	0.15	0.69	0.78	0.05
	3	0.74	0.26	0.01	0.19	0.72	0.09	0.04	0.14	0.82	0.76	0.04
	4	0.94	0.05	0.01	0.16	0.72	0.12	0.05	0.36	0.59	0.75	0.12
	Mean	0.84	0.13	0.03	0.14	0.76	0.10	0.09	0.21	0.70	0.76	0.05
	Std	0.05	0.06	0.02	0.03	0.04	0.02	0.05	0.07	0.06	0.05	0.01
FS-IF	1	0.87	0.12	0.02	0.12	0.75	0.13	0.06	0.15	0.79	0.80	0.04
	2	0.88	0.05	0.06	0.09	0.85	0.05	0.09	0.06	0.85	0.86	0.01
	3	0.87	0.12	0.01	0.17	0.76	0.06	0.04	0.11	0.86	0.83	0.05
	4	0.95	0.04	0.01	0.13	0.68	0.20	0.05	0.33	0.62	0.75	0.14
	Mean	0.89	0.08	0.02	0.13	0.76	0.11	0.06	0.16	0.78	0.81	0.05
	Std	0.03	0.04	0.02	0.02	0.05	0.05	0.02	0.09	0.08	0.05	0.02
IA-IF	1	0.86	0.10	0.04	0.12	0.74	0.14	0.08	0.15	0.76	0.79	0.05
	2	0.88	0.09	0.03	0.09	0.82	0.09	0.11	0.08	0.81	0.84	0.03
	3	0.81	0.16	0.03	0.21	0.70	0.10	0.06	0.15	0.79	0.77	0.05
	4	0.96	0.04	0.01	0.11	0.69	0.20	0.05	0.33	0.62	0.76	0.13
	Mean	0.88	0.10	0.03	0.13	0.74	0.13	0.08	0.18	0.75	0.79	0.06
	Std	0.04	0.03	0.01	0.04	0.04	0.04	0.02	0.08	0.06	0.05	0.01
FS-IA-IF	1	0.86	0.12	0.02	0.13	0.75	0.12	0.06	0.16	0.78	0.80	0.04
	2	0.88	0.09	0.03	0.10	0.82	0.09	0.08	0.12	0.81	0.84	0.03
	3	0.77	0.20	0.02	0.24	0.66	0.10	0.04	0.17	0.78	0.74	0.05
	4	0.96	0.04	0.01	0.12	0.71	0.17	0.03	0.35	0.62	0.76	0.13
	Mean	0.87	0.11	0.02	0.15	0.73	0.12	0.05	0.20	0.75	0.78	0.06
	Std	0.05	0.05	0.01	0.05	0.05	0.02	0.02	0.07	0.06	0.05	0.01

Success rate for the classification set considering each preprocessing method and studied task. Results are normalized between 0 and 1, in which 1 means 100%. Actual and predicted (between brackets) classes are shown as headers of the table, for instance, S_H_(S_PD_) means that the actual class is S_H_ and the predicted class was S_PD_. MeanTP and StdTP are respectively the mean and standard deviation of true positives, i.e., S_H_(S_H_), S_PD_(S_PD_) and S_DBS_(S_DBS_). The mean and standard deviation (Std) are presented

### Amplitude features

MAV: Mean absolute value;
RMS: Root mean squared deviation;
PEAK: Global maximum;
MAVSDN: Mean of the absolute values of the second differences of the normalized signal;
MAVSD: Mean of the absolute values of the second differences;
MAVFDN: Mean of the absolute values of the first differences of the normalized signal;
MAVFD: Mean of the absolute values of the first differences of the signal

### Variability features

INTERQ_RANGE: Interquartile range of the signal;
RANGE: Difference between the maximum and minimum values of a signal;
STD: Standard deviation;
VAR: Variance

### Entropy

Approximate entropy—it measures the number of specific ways in which a system may be arranged, often taken to be a measure of disorder.

For each method (i.e., FS, IA and IF), a feature matrix was created containing the features extracted from all sensors. In addition, it was analyzed the combination of features estimated from each method: FS–IA, FS–IF, IA–IF, FS–IA–IF. The aim was to identify which combination could provide us with the best discrimination results.

In order to reduce the dimensionality of the feature space to a two-dimensional space, the Sammon’s mapping method was used (step 7 in Fig. 4). The Sammon’s algorithm maps a high-dimensional space and converts it to a space of lower dimensionality, trying to preserve the structure of inter-point distances in high-dimensional space in the lower-dimension projection [43]. The selection of Sammon’s map is based on the relative success of this method for dealing with nonlinearities inherent to our data type [44].

Data projections were carried out for each specific task and then a scatter plot of the obtained projection was generated (step 8 in Fig. 4), so that possible differences among the studied groups could be visualized.

The analysis of the lower dimensional data was performed by means of two distinct strategies: (i) the use of statistical analysis; (ii) the evaluation of classification results.

### Statistical analysis

For the statistical evaluation of the data (step 9 in Fig. 4), the non-parametric analysis of variance method (NPMANOVA—non-parametric MANOVA) was applied. This is because the bivariate normality presupposition was not satisfied for any of the tasks. The bivariate normality presupposition was verified by means of the Mardia test [45]. But, at the significance level of 5%, the bivariate normality was not successful with any of the tasks.

The NPMANOVA, also known as PERMANOVA (permutational multivariate analysis of variance) is widely used for ecological data. The test consists in comparing measurements of distances between observed pairs within the same group versus distances in different groups, so it is a nonparametric test that compares distance between two or more groups by means of distance measurements [46]. In the NPMANOVA analysis it was used the Euclidean distance, considering 10,000 permutations. This analysis was performed in the software PAST (PAleontological STatistics) [47] with a confidence level of 95%. The Bonferroni’s correction was applied. It was performed by multiplying the p values by the number of paired comparisons of the values obtained from each feature matrix (FS, IA, IF, FS–IA, FS–IF, IA–IF and FS–IA–IF).

### Classification analysis

In addition to the statistical analysis, K-fold cross-validation for discriminant analysis based on Gaussian finite mixture modeling [48] was employed for data classification (step 10 in Fig. 4). For this the toolbox *mclust* (available in R Project for Statistical Computing [49]) [50] was used. The eigenvalue decomposition discriminant analysis (EDDA) [48] model and a K of 10 were employed. The low dimensional data set (2D) was used as input for the model.

The training and test sets were created randomly, by selecting data from five participants for each group (S_H_, S_PD_, S_DBS_), and without repetition (i.e., data from individuals of the training set were not used in the test set) in order to validate the classifier and avoid overfitting. In total, 1000 pairs of training and test sets were created.

The accuracy of the classifier was measured by means of the normalized success rate (0–1), which is the number of correctly classified patterns of a class over the total number of patterns of this class.

## Results

### Visualization and statistical results of projected data

**Fig. 6:**
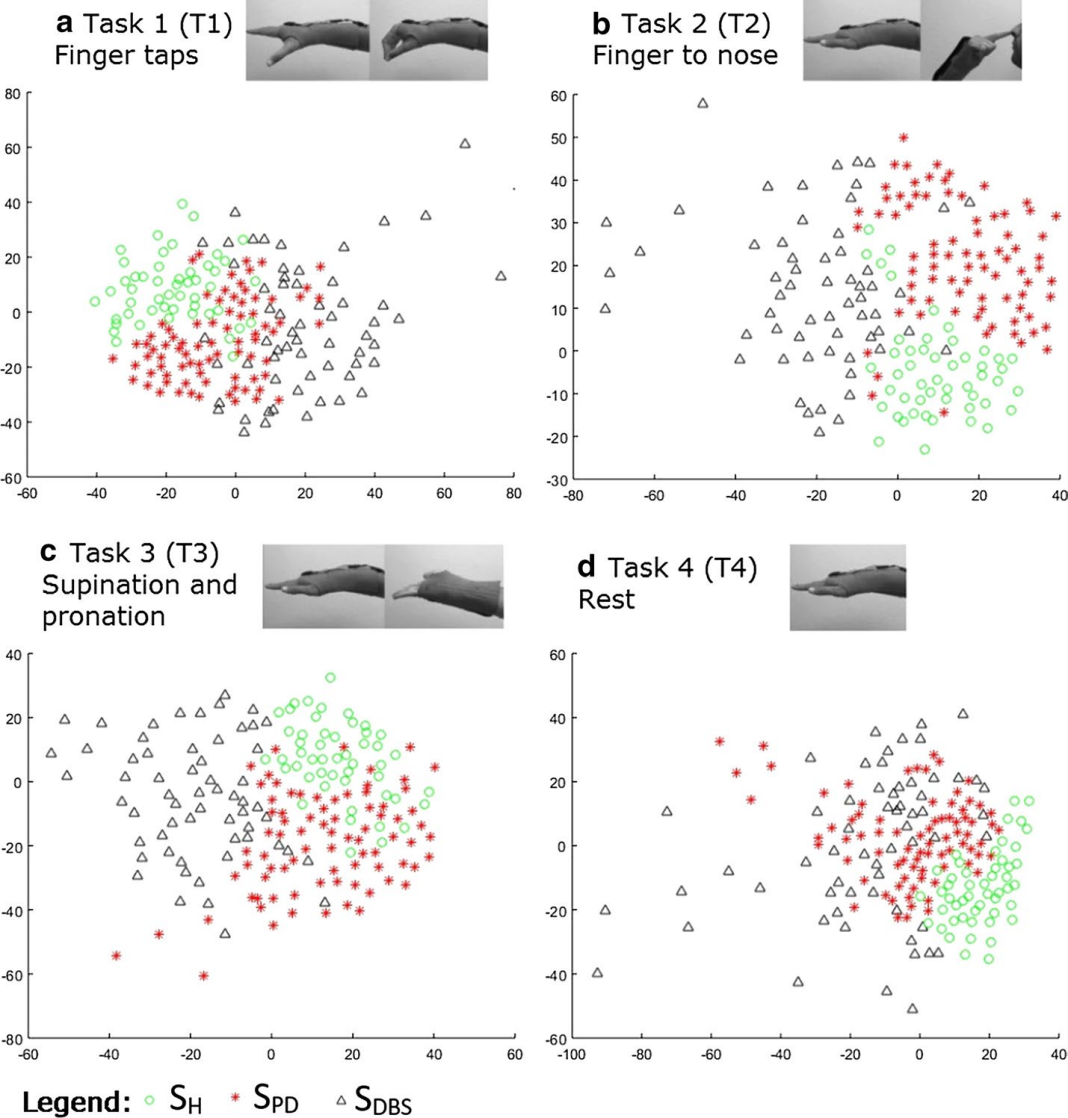
Visualization of projected data onto a lower dimensional space. Separation of groups by the use of the Sammon’s projection method. The visualizations are scatter plots representing dimensionless scores of the nonlinear projection of high-dimensional feature vectors (FS-IA) onto the first (*x axis*) against the second (*y axis*) estimated components. *Triangles* represent SDBS, *circles* SH and *asterisks* SPD. **a** represents Task 1, **b** Task 2, **c** Task 3 and **d** Task 4

Figure 6 depicts an example of data projection and dimensionality reduction obtained by the combination of features extracted from the FS and IA (FS-IA) methods. In the figure, it is possible to observe the separation between groups. Results for each task are shown in A (Task 1), B (Task 2), C (Task 3) and D (Task 4). In the figure the triangles represent S_DBS_, circles represent S_H_ and asterisks represent S_PD_.

Visually there was a clear separation among groups for all tasks and feature combinations. For all the tasks and methods, the *p* value with Bonferroni’s correction was lower than 0.05. This statistically confirmed the discrimination between the three groups.

### Classification results of projected data

Table 1 presents the success rate (normalized between 0 and 1) for the data (i.e., classification data set) employed for *training* the classifier, whereas Table 2 shows the results of new data (i.e., test data set) presented to the *trained* classifier.

**Table 2:** Success rate for the test set

Test												
Method	Task	S_H_ (S_H_)	S_H_ (S_PD_)	S_H_ (S_DBS_)	S_PD_ (S_H_)	S_PD_ (S_PD_)	S_PD_ (S_DBS_)	S_DBS_ (S_H_)	S_DBS_ (S_PD_)	S_DBS_ (S_DBS_)	MeanTP	StdTP
FS	1	0.73	0.18	0.08	0.15	0.67	0.17	0.14	0.21	0.65	0.69	0.03
	2	0.68	0.15	0.17	0.15	0.72	0.13	0.22	0.17	0.61	0.67	0.04
	3	0.59	0.31	0.10	0.25	0.56	0.19	0.15	0.24	0.61	0.59	0.02
	4	0.73	0.20	0.08	0.27	0.48	0.24	0.07	0.40	0.53	0.58	0.10
	Mean	0.68	0.21	0.11	0.21	0.61	0.18	0.14	0.26	0.60	0.63	0.03
	Std	0.05	0.05	0.03	0.06	0.09	0.03	0.04	0.07	0.04	0.06	0.02
IA	1	0.72	0.17	0.11	0.14	0.69	0.17	0.18	0.21	0.61	0.67	0.04
	2	0.65	0.25	0.10	0.23	0.63	0.13	0.21	0.14	0.65	0.64	0.01
	3	0.62	0.33	0.06	0.30	0.53	0.16	0.12	0.20	0.68	0.61	0.05
	4	0.77	0.11	0.12	0.14	0.61	0.26	0.12	0.40	0.47	0.62	0.10
	Mean	0.69	0.22	0.10	0.20	0.62	0.18	0.16	0.24	0.60	0.64	0.03
	Std	0.06	0.07	0.02	0.06	0.05	0.04	0.04	0.08	0.06	0.06	0.01
IF	1	0.82	0.13	0.05	0.22	0.47	0.31	0.13	0.29	0.58	0.62	0.13
	2	0.85	0.07	0.09	0.16	0.66	0.18	0.13	0.15	0.72	0.74	0.07
	3	0.72	0.21	0.07	0.27	0.49	0.24	0.13	0.18	0.69	0.63	0.09
	4	0.89	0.08	0.03	0.15	0.49	0.36	0.15	0.34	0.52	0.63	0.17
	Mean	0.82	0.12	0.06	0.20	0.53	0.27	0.13	0.24	0.63	0.66	0.11
	Std	0.05	0.05	0.02	0.04	0.07	0.06	0.01	0.07	0.08	0.07	0.01
FS-IA	1	0.74	0.17	0.09	0.15	0.67	0.18	0.15	0.23	0.62	0.68	0.04
	2	0.68	0.19	0.14	0.19	0.67	0.14	0.19	0.18	0.62	0.66	0.02
	3	0.65	0.32	0.03	0.28	0.55	0.17	0.10	0.19	0.71	0.64	0.06
	4	0.76	0.16	0.09	0.19	0.55	0.26	0.06	0.41	0.53	0.61	0.10
	Mean	0.71	0.21	0.09	0.20	0.61	0.19	0.13	0.25	0.62	0.65	0.04
	Std	0.04	0.06	0.03	0.04	0.06	0.04	0.05	0.08	0.05	0.05	0.01
FS-IF	1	0.81	0.16	0.03	0.19	0.61	0.20	0.08	0.22	0.70	0.71	0.07
	2	0.85	0.07	0.00	0.13	0.77	0.09	0.14	0.10	0.76	0.79	0.04
	3	0.79	0.19	0.02	0.22	0.65	0.13	0.08	0.15	0.78	0.74	0.06
	4	0.82	0.13	0.05	0.16	0.52	0.33	0.07	0.44	0.49	0.61	0.14
	Mean	0.82	0.14	0.03	0.18	0.64	0.19	0.09	0.23	0.68	0.71	0.07
	Std	0.02	0.04	0.02	0.03	0.07	0.07	0.02	0.11	0.10	0.06	0.03
IA-IF	1	0.80	0.15	0.05	0.17	0.63	0.20	0.10	0.22	0.67	0.70	0.07
	2	0.82	0.13	0.05	0.13	0.74	0.13	0.15	0.13	0.72	0.76	0.04
	3	0.74	0.21	0.05	0.27	0.57	0.16	0.11	0.19	0.70	0.67	0.06
	4	0.82	0.14	0.05	0.14	0.55	0.31	0.06	0.44	0.49	0.62	0.13
	Mean	0.79	0.16	0.05	0.18	0.62	0.20	0.11	0.25	0.65	0.69	0.07
	Std	0.03	0.03	0.00	0.05	0.06	0.06	0.02	0.10	0.08	0.06	0.02
FS-IA-IF	1	0.78	0.18	0.04	0.18	0.64	0.18	0.08	0.21	0.71	0.71	0.05
	2	0.82	0.12	0.05	0.14	0.71	0.15	0.13	0.00	0.72	0.75	0.05
	3	0.70	0.25	0.04	0.31	0.53	0.16	0.09	0.21	0.70	0.64	0.08
	4	0.80	0.15	0.06	0.16	0.55	0.29	0.06	0.44	0.50	0.62	0.12
	Mean	0.78	0.18	0.05	0.20	0.61	0.20	0.09	0.22	0.66	0.68	0.06
	Std	0.04	0.04	0.01	0.06	0.07	0.05	0.02	0.11	0.08	0.06	0.02

Success rate for the test set considering each preprocessing method and studied task. Results are normalized between 0 and 1, in which 1 means 100%. Actual and predicted (between brackets) classes are shown as headers of the table, for instance, S_H_(S_PD_) means that the actual class is S_H_ and the predicted class was S_PD_. MeanTP and StdTP are respectively the mean and standard deviation of true positives, i.e., S_H_(S_H_), S_PD_(S_PD_) and S_DBS_(S_DBS_). The mean and standard deviation (Std) are presented

### Tasks

Task 2 (finger to nose) had the highest success rate (overall mean 0.80 and 0.72, respectively for the classification and test sets). Task 3 (supination and pronation) presented the worst overall success rate (0.71, for the classification set) while Task 4 (rest) showed the worst overall success rate (0.66) for the test set. Task 2 yielded the lowest overall mean standard deviation for the classification and test sets, respectively, 0.04 and 0.04. The largest overall mean standard deviation was for Task 4 (0.13 and 0.12, for classification and test sets, respectively).

### Preprocessing methods

Considering the methods, the preprocessing method FS–IF was the one which yielded the highest success rates (overall mean respectively 0.81 and 0.71 for the classification and test sets), and the methods FS and IF showed the worst success rates for classification sets (0.74) and FS showed the worst success rate for test sets (0.63). Also, it could be observed that for Tasks 1, 2 and 3, the best method was FS–IF for both classification (0.80, 0.86 and 0.83, respectively) and test (0.71, 0.79 and 0.74, respectively) sets, and for Task 4 the best method for classification was IA-IF and FS-IA-IF (0.76) and the best method for test was IF (0.63) sets. The highest overall standard deviation was obtained from the method IF, for both classification and test sets (0.10 and 12 respectively).

### Discrimination among groups

S_H_ showed the highest success rates for the classification set for FS–IF (0.89) and the highest success rates for the test set for IF and FS–IF (0.82). S_PD_ showed the highest success rates for the classification for IA, FS-IA and FS–IF (0.76) and the highest success rates for the test set for FS–IF (0.64). Finally, S_DBS_ showed the highest success rate for the classification and test sets for FS–IF (0.78 and 0.68, respectively).

S_DBS_ group showed the worst mean success rate for tasks 1, 2 and 4 (0.72, 0.77 and 0.60, respectively) compared to S_H_ (0.85, 0.85 and 0.94, respectively) and S_PD_ (0.74, 0.81 and 0.69, respectively). Only for Task 3, S_PD_ showed the lowest success rate compared to other groups (0.77—S_H_, 0.69—S_PD_ and 0.79—S_DBS_).

Considering the mean values of all the methods and for each task in S_H_, Task 4 yielded the highest success rate for the classification and test sets (0.94 and 0.80, respectively) and Task 3 yielded the worst success rate for the classification and test sets (0.77 and 0.69, respectively). For S_PD_, Task 2 yielded the highest success rate for the classification and test sets (0.81 and 0.70, respectively) and Tasks 3 and 4 yielded the worst success rate for the classification set (0.69) and Task 4 the worst success rate for the test set (0.54). Finally, for S_DBS_, Task 3 yielded the highest success rate for the classification set (0.79) and Tasks 2 and 3 showed the highest success rates for the test sets (0.69) and Task 4 yielded the worst success rate for the classification and test sets (0.60 and 0.50, respectively).

## Discussion

Consistent with the literature [12, 22, 23, 27–33] our results demonstrated differences between movement patterns for the three groups. We however introduce the possibility of visualizing and classifying the data obtained from subjects objectively, independent of the experience of examiners and subjective rating scales. Based on our review, this is the first study in this direction.

From the proposed method for data analysis, several parameters could be extracted from individual’s data. Parameters regarding the tasks, preprocessing methods and subjects provided important information regarding specific characteristics of groups of individuals and treatments.

Our results take in account the differentiation of PD treatments and a healthy control group without considering the subtypes of the disease. It is not known whether the existence of these subtypes of the disease have generated any influence over our results, since tremor, bradykinesia and rigidity present different movement patterns. The variability found in some methods may be due to this factor. Also, there is the possibility of different behaviors in the execution of the tasks provided by the subtypes. A further study, with the use of our system and protocol in new groups of participants, separated by PD subtypes, could address this issue.

### The relevance of the tasks

From a methodological perspective, the tasks performed in this study are well established, described in the UPDRS [18] and used in clinical evaluation. Several studies evaluated finger taps (Task 1) [51–55], finger to nose task (Task 2) [51], pronation and supination (Task 3) [56], and postural tremor of the hands (Task 4) [57]. Thus, results from our study came from real procedures widely used in neurological assessments.

According to the visualization obtained from the Sammons mapping technique, the groups could be discriminated while executing the distinct experimental tasks. These results confirm the discriminations already observed in subjective evaluations and, additionally, confirm that the executed tasks allow for the discrimination between groups and types of treatment. Further detailed studies will be required both within subjects and across conditions and with precise comparisons with clinical scales to further validate this approach.

The classification results were presented in order to support the visual information provided by the Sammon’s map projection. The classification analysis allows for the evaluation of models for the groups, which are generated through the available experimental data. In order to analyze the generalization of the model, i.e., results based on data sets not used for the model estimate, we divided our data sets into classification and test sets. By taking into account this we were able to obtain the results shown in Tables 1 and 2, from which it was possible to objectively understand differences among the groups, tasks and preprocessing methods.

The task that yielded the best success rate was Task 2 (finger to nose). For this task the overall mean success rate was of 0.80 and 0.72, for the classification and test sets, respectively. When compared to the other tasks, Task 2 is considerably more complex and with the largest movement extension, as it involves the coordination of the arm, forearm and shoulder. However, this motor complexity seems to generate data patterns that best characterize (i.e., yields less overlapping between groups) the studied groups as confirmed by the success rates shown in Tables 1 and 2.

Still, the relatively high success rates (above 0.75 and 0.61 for the classification and test sets, respectively) obtained for the other tasks indicate they cannot be neglected for discrimination purposes. Future studies should consider the joint analysis of features extracted from distinct types of tasks, with the aim of improving success rates.

Although the four tasks demonstrated good results regarding group separation, other tasks, which are part of the clinical routine, can also be analyzed in the future.

### The role of the preprocessing method

In this study, three preprocessing methods were employed. The first (FS) was based on the filtered signal, which yields data more correlated with the original data; the second (IA) takes into account changes in the amplitude of the signal; and the third (IF) captures changes in the signal frequency over time.

From our results, it is possible to conclude that the combination (i.e., joint analysis of features extracted from the methods FS and IF) was the one that yielded the best overall success rate. When considering Task 2 and FS–IF, we report a success rate of 0.86 and 0.79 for the classification and test sets, respectively. The success of this method (when compared to the IA and its combinations) may be related to the considerably high overlap between amplitude components yielded by the execution of distinct tasks.

### The overall evaluation of the success rates

A summary of the overall mean success rates and their standard deviation (estimated from Tables 1, 2) for the true positive predictions are given below:

- S_H_ (S_H_): 0.85 ± 0.05 and 0.75 ± 0.04 for classification and test sets, respectively;
- S_PD_ (S_PD_): 0.73 ± 0.05 and 0.60 ± 0.07 for classification and test sets, respectively;
- S_DBS_ (S_DBS_): 0.72 ± 0.06 and 0.63 ± 0.07 for classification and test sets, respectively.

From Tables 1 and 2 it is also possible to estimate the overall degree of overlapping between groups (and its standard deviation) as shown below:

- S_DBS_ (S_PD_): 0.19 ± 0.07 and 0.24 ± 0.09 for classification and test sets, respectively;
- S_PD_ (S_DBS_): 0.12 ± 0.03 and 0.20 ± 0.05 for classification and test sets, respectively;
- S_H_ (S_PD_): 0.11 ± 0.05 and 0.17 ± 0.05 for classification and test sets, respectively;
- S_PD_ (S_H_): 0.14 ± 0.04 and 0.20 ± 0.05 for classification and test sets, respectively;
- S_H_ (S_DBS_): 0.04 ± 0.02 and 0.07 ± 0.02 for classification and test sets, respectively;
- S_DBS_ (S_H_): 0.09 ± 0.03 and 0.12 ± 0.03 for classification and test sets, respectively.

S_PD_ showed overlapping with the two other groups. This may be related to the variability of the results of the treatment (levodopa), which is time-dependent, and thus can yield a larger variability of motor patterns.

The inter-group variability was low (less than 0.07) suggesting similarities of motor patterns of individuals within the same group.

### Potential and practical applications

The results found in this study can be employed in distinct contexts. The data visualization shown in Fig. 6 and the boundaries for distinct groups (which is essentially created by the classification method) could be considered as typical motor patterns for each group, projected onto a low dimensional space. The main limitation of our study is the relatively low number of participants, however as this number increases the reliability of the model also increases, and then the better this model could represent the actual world, by taking into account the inherent variability of inertial and electromyographic data.

In many circumstances, the diagnosis of PD is not straightforward, thus the discrimination between healthy control and affected subjects is beneficial for both initial diagnosis and the management of disease progression.

In the worst scenario, when the disease cannot be managed by the use of medication, or when this medication interferes with the quality of life of patients, the current medical guidelines suggest consideration of surgical intervention. Regarding DBS, the consensus is to provide the surgery to PD patients when levodopa fails to provide consistent benefit and all other options have not been successful. Taking this information into account, Xie et al. [23] suggested that in order to evaluate the best moment for the DBS implant surgery, more studies should be performed. Furthermore, the expectation of improving the quality of life of patients treated with DBS has to be weighed against the risk of some serious complications related to surgery [22]. Adverse effects of DBS consist in hemorrhage resulting in permanent neurological deficit or death in 1.1%, infections, electrode migrations or misplacements, skin erosion, wire fractures and device malfunction. The rate of appearance of these complications are between 4.3 and 17.8% [23]. In general, DBS is a relatively safe approach, but not free from risks. By using the visualization tools such as that in Fig. 6 one could objectively monitor the progress of the disease, by comparing motor patterns of the patients with that of groups of people with PD. A more important point is that such a visualization of data points is able to show whether the patient is leaving the area of medication treated patients, and this could be an evidence of motor deterioration, which could be used in the decision of recommendation for DBS.

An innovative aspect of our research is certainly the inclusion of individuals with DBS implant. By doing this we were able to visualize motor patterns of these individuals, which has not been reported in the literature. The specific motor patterns of DBS users should be further studied and explained, by synchronizing information from the stimulator, inertial, electromyographic and electrocorticographic signals.

Data from more patients treated with medication can be included in the database and thus, further studied. The use could be straightforward: when the subject is not classified in the expected group clinicians should verify whether the proposed treatment is as effective as it should be. In such a scenario, different management schemes could be pursued in order to optimize patient management and care.

## Conclusions

In conclusion, the device and methods described in this study could potentially improve upon current management algorithms for patients with PD, potentially limiting the role of subjective methods and scales that are sensitive and therefore limited by human bias. The visualization provided by the Sammon’s map preserves the distance between groups, so that they could be clearly identified for all investigated tasks. The preprocessing method based on the combination of filtered signals with their instantaneous amplitude (FS–IF) was the one that provided the best success rates, being the most effective in discriminating the three studied groups. The static task (Task 4) allowed for the best discrimination of healthy individuals. Task 2 (finger to nose) allowed for the best discrimination of PD subjects and Task 3 (supination and pronation) showed the best discrimination of DBS subjects.

## Abbreviations

DBS: deep brain stimulation
PD: Parkinson’s disease
UPDRS: Unified Parkinson’s Disease Rating Scale
EMG: electromyography
